# Improved cardiac auscultation competency interweaving visual, auditory, and tactile stimuli: a preliminary study

**DOI:** 10.5116/ijme.6607.27a6

**Published:** 2024-04-05

**Authors:** Harrison A. Patrizio, Riley Phyu, Bum Kim, Nils V. Brolis

**Affiliations:** 1 Rowan University School of Osteopathic Medicine, USA; 2 Department of Family Medicine at Rowan University School of Osteopathic Medicine, USA

**Keywords:** Cardiac auscultation, education, simulation, competency

## Abstract

**Objectives:**

To assess the efficacy of a cost-effective, multisensory teaching approach for improving cardiac auscultation competencies among medical students to combat the global decline in cardiac auscultation skills.

**Methods:**

A pilot randomized controlled trial was conducted at our institution’s simulation center with 32 first year medical students from a single medical institution. Participants were randomly divided into two equal groups and completed an educational module the identification and pathophysiology of five common cardiac sounds. The control group utilized traditional education methods, while the interventional group incorporated multisensory stimuli. Afterwards, participants listened to randomly selected cardiac sounds and competency data was collected through a multiple-choice post-assessment in both groups. Mann-Whitney U test was used to analyze the data.

**Results:**

Data were analyzed using the Mann-Whitney U test. Diagnostic accuracy was significantly higher in the multisensory group (Mdn=100%) compared to the control group (Mdn=60%) on the post-assessment (U=73.5, p<0.042). Likewise, knowledge acquisition was substantially better in the multisensory group (Mdn=80%) than in the control group (Mdn=50%) (U= 49, p<0.031).

**Conclusions:**

These findings suggest the incorporation of multisensory stimuli significantly improves cardiac auscultation competency. Given its cost-effectiveness and simplicity, this approach offers a viable alternative to more expensive simulation technologies like the Harvey simulator, particularly in settings with limited resources. Consequently, this teaching modality holds promise for global applicability, addressing the worldwide deterioration in cardiac auscultation skills and potentially leading to better patient outcomes. Future studies should broaden the sample size, span multiple institutions, and investigate long-term retention rates.

## Introduction

Cardiac auscultation is a fundamental skill in medicine that enables healthcare professionals to identify potential cardiac pathologies and initiate prompt treatment.^1^ As the prevalence of outpatient physician encounters with reduced access to immediate cardiac imaging continue to increase, auscultation skills remain very relevant for patient care.^1^ Despite its critical importance, studies have shown that the proficiency of medical professionals in performing cardiac auscultation is in a steady decline.^2^^-^^4^This decline is particularly evident in a study by Mangione and colleagues that showed only 20% of the graduating medical class could accurately diagnosis cardiac sounds. They concluded that this result may be largely due to curriculum deficiencies in teaching cardiac auscultation.^5^ This is supported by a follow up study finding a similar competency level even after students gain more exposure in residency, suggesting that clinical experience is also not the solution.^5^

Traditional methods of teaching cardiac auscultation involve employing teaching methods, such as 2D static slide-based education for cardiac anatomy and audio recordings for memorization of cardiac sounds.^6^However, these methods have limitations since it requires students to be able to actually depict anatomical relationships and have spatial understanding of interconnected body systems.^7^ Additionally, isolated audio recordings rely on brute memorization and may not resemble the variety found in real life. The use of 3D animated videos have tried to eliminate this issue by adding more dimension and visual representation on pathophysiology, however this method has not been shown to be as effective as the use of expensive physical simulators given the lack of learner engagement.^8^^-^^10^Simulation mannequins, such as Harvey, have been developed to address these issues. However, their high cost, at nearly $100,000 each, limits accessibility for many programs.^11^^,^^12^ Furthermore, even when programs have the funds to purchase them, one study found that only 25-48% of Internal Medicine Residency programs actually provided their students with instruction using these mannequins.^5^^,^^6^ This may be partly due to simulator availability, given the larger institutions capable of purchasing these tools may need to divide time across multiple programs.^13^ Therefore, expensive simulators cannot always be relied on for auscultation education.

Considering the mixed research findings, there is a need for an easily implemented, cost-effective, and engaging alternative to current teaching methods in cardiac auscultation. Multisensory learning theory, which integrates multiple senses to reduce cognitive load and enhance recall accuracy, presents a promising solution to these challenges.^14^ This approach has demonstrated improved learning outcomes in various studies. For instance, Nyberg and colleagues found that participants who received visual and auditory stimuli together, achieved higher recognition scores compared to those exposed to visual stimuli alone.^15^ In addition to visual and auditory stimuli, incorporating tactile senses can further enhance learning experiences by fostering engagement and improving learning outcomes. One example by Ginns and colleagues revealed that students who traced their index finger over mathematical diagrams during instruction committed fewer errors than those who did not, underscoring the effectiveness of integrating tactile senses into the learning process.^16^ By incorporating multisensory learning theory into the curriculum through minor adjustments to existing programs, it may be possible to address the limitations of current teaching methods in cardiac auscultation education.

Our preliminary study seeks to investigate the potential of this novel educational design that combines visual, auditory, and tactile stimuli to enhance the understanding and diagnostic accuracy of cardiac sounds, while maintaining low entry costs and student engagement. We hypothesize, based on the previous literature, that integrating multisensory learning theory into the current curriculum will bridge the gap between traditional teaching methods and expensive simulation tools, ultimately improving the proficiency of medical professionals in cardiac auscultation and leading to better patient outcomes.^15^^,^^16^

## Methods

### Study Design, Sample Size, and Participants

After the Institutional Review Board (IRB) of the Rowan School of Osteopathic Medicine granted ethical approval, in adherence to the Helsinki Declaration guidelines, a pilot randomized control was performed to assess the competency of recognizing heart sounds, comparing the effectiveness of multisensory learning in the interventional group with traditional methods used in the control group. The study took place in the simulation center at the Rowan School of Osteopathic Medicine, providing an academic environment for the research. Ethical considerations were taken into account in this study to ensure the safety, privacy, and well-being of the participants. Informed consent was obtained from all participants prior to their involvement in the study, with a clear explanation of the research objectives and procedures. Confidentiality was maintained throughout the study by anonymizing participant data and securely storing information.

All incoming first-year medical students enrolled during the summer of 2022 (n=56) were invited to participate in the study. All students received an invitation flyer with a study overview and a pre-assessment survey link. The pre-assessment survey included basic information such as experience with cardiac auscultation, perceived understanding of heart sounds, and confidence in identifying heart sounds. Inclusion criteria were no previous cardiac auscultation skills training or clinical experience with auscultation. Students with prior healthcare experience using stethoscopes, such as physician assistants and nurses, were excluded.

**Figure 1.**
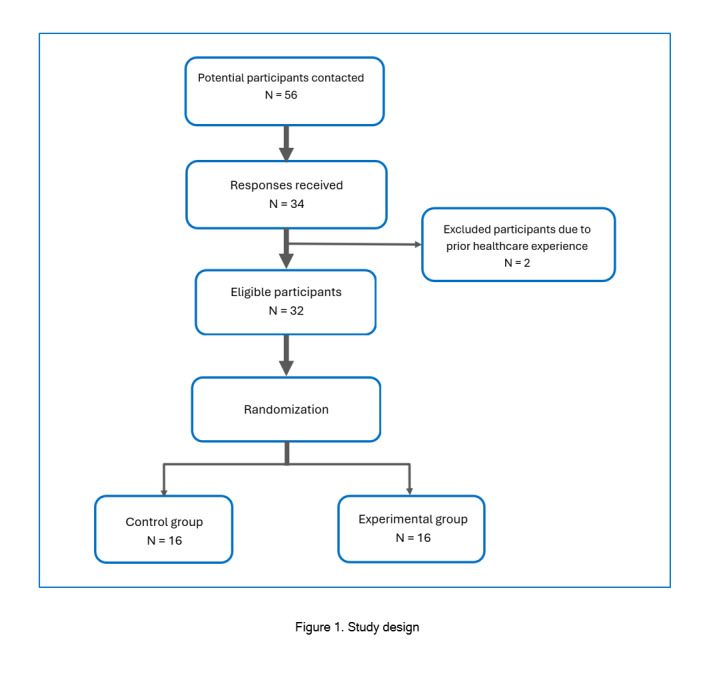
Study design

Of the 56 surveyed students, 34 (response rate = 60.7%) completed the survey. The participants were a diverse group of first-year medical students with various educational backgrounds, consisting of 18 females and 16 males, all interested in pursuing a medical degree. After review, two students were excluded due to previous cardiac auscultation experience, leaving a sample size of 32 participants for this study. After giving informed consent, the 32 participants were randomly assigned to either the control or interventional groups using a random number generator (Figure 1). The study was voluntary, and participants were not compensated. All documents were de-identified and matched using anonymous codes based on seating location in the assessment area.

### Interventions

In a conference room setting, control group (n=16) received a 10-minute pre-recorded slide presentation comprising 2D phonocardiogram visualization with narration and overlaid heart sounds, which are traditionally used modalities for teaching cardiac auscultation. The interventional group (n=16) received the same 10-minute pre-recorded slide presentation, supplemented with simulated cardiac cycles of 3D cross-sectioned hearts and haptic synchronization.

Vimedix, a virtual human anatomy simulator, provided the 3D cross-sectional cardiac cycles. Heart sounds recorded from Harvey, the cardiopulmonary simulator, were overlaid on each cardiac cycle clip and on the respective 2D phonocardiograms presented in the control group's video. Haptic synchronization involved participants tapping along with the heart sounds played in the video when the notification appeared on screen. Printed phonocardiograms were provided for each heart sound as a tapping guide. These components created a multisensory environment incorporating visual, auditory, and tactile learning in the educational material.

Both groups were taught five different heart sounds: S1/S2, S3, S4, crescendo-decrescendo, and mid-systolic click. These heart sounds were chosen because they are associated with common cardiac sounds.^1^ Each group's video included associated heart sounds and an explanation of their pathophysiologies.^1^

### Data Collection and Outcome Measures

**Figure 2.**
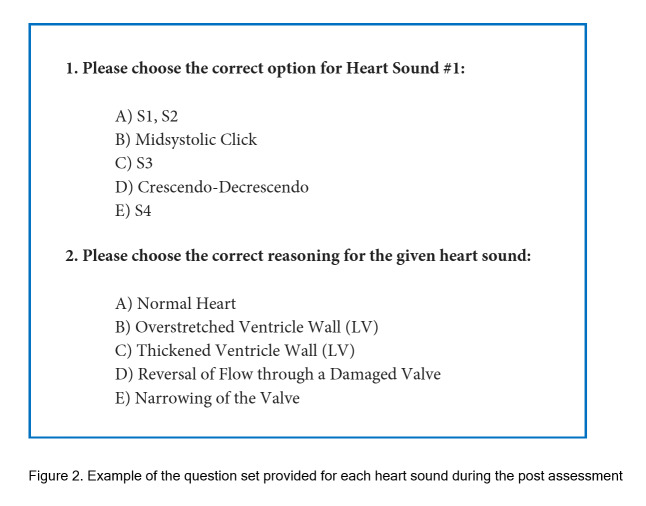
Example of the question set provided for each heart sound during the post assessment

Participants completed a post-assessment multiple-choice exam to evaluate their overall competency approximately 10 minutes after training due to the transitional period. During the transition period, participants were moved from the training room to a pre-setup assessment room and then were given instructions to complete the post assessment to the best of their ability. The assessment comprised the five different heart sounds presented in a randomized order through wireless stethoscopes to better approximate real-world conditions. The wireless stethoscopes also allowed us to test all participants at the same time to avoid variability in time assessed after training. Overall competency was divided into two distinct categories: the ability to correctly identify heart sounds, diagnostic accuracy, and the ability to explain the underlying pathophysiology, knowledge acquisition. For each heart sound, participants were asked to answer one diagnostic accuracy question and one knowledge acquisition question, resulting in a total of ten questions. These questions were presented in an online form to reduce grading time and improve grading accuracy by removing the human component. An example of these questions can be found in Figure 2. No feedback was given to the students during or after the assessment.

Accurately answering a question earned the student one point for that question. As there were five heart sounds, each with one question pertaining to diagnostic accuracy and another to knowledge acquisition, the total score for both categories was calculated individually for each participant on a scale from 0 to 5. A score of 0 signified that no questions were answered correctly, whereas a score of 5 indicated that all questions in the respective category were answered accurately. For example, answering all 5 diagnostic accuracy questions would yield a 5 out of 5 score, but getting one question incorrect would yield a score of 4 out of 5. This same format was applied to knowledge acquisition.

To ensure the validity of the post-assessment test, the questions were developed based on published educational handbooks that provide detailed descriptions of each heart sound.^1^ Additionally, experienced clinicians and educators in the field were consulted to review the questions for content validity and ensure they accurately reflected the key aspects of cardiac auscultation and pathophysiology. We used this style of assessment because it has shown to be useful in other studies such as Barrett and colleagues that assessed participants using 10 different heart sounds in a randomized order and graded them using clinicians as support.^17^

#### Analysis

All statistical tests were performed using Microsoft Excel 2021 by HP and RP. The Shapiro-Wilk test was first conducted to test for normality. Due to the non-normal distribution of the data, a nonparametric Mann-Whitney U test with a 95% confidence interval was used to analyze statistical differences in diagnostic accuracy and knowledge acquisition scores between the control and interventional groups. The effect size was calculated using rank-biserial correlation test to determine the magnitude of differences between the groups. Effect sizes were measured using the rank-biserial correlation coefficient and were categorized based on the absolute value of this coefficient. The categories were defined as: very weak for values between 0.00 and 0.19, weak for values from 0.20 to 0.39, moderate for values ranging from 0.40 to 0.59, strong for values between 0.60 and 0.79, and very strong for values from 0.80 to 1.00.^18^

## Results

The variables under analysis were the ability to correctly identify heart sounds (diagnostic accuracy) and the ability to explain the pathophysiology (knowledge acquisition) in both the control and interventional groups. Mean, standard deviation, mean of ranks, effect size (rank-biserial correlation) is provided in Table 1.

### Diagnostic Accuracy

All 32 participants completed the post assessment exam for diagnostic accuracy. The average score in the control group was 58.75% (SD = 1.77) and the average score was 85% (SD = 0.93) in the interventional group. (Table 1) Due to our small sample size, determining the distribution of the scores was important for choosing the statistical method. The Shapiro-Wilks test was performed and showed that the distribution of scores deviated significantly from normality in the control (W= 0.87, p-value < 0.04) and interventional groups (W= 0.70, p-value < 0.0002). Due to the substantial skewness of the data, a non-parametric Mann-Whitney U test was used for analysis. The analysis indicated that diagnostic accuracy was significantly improved for multisensory students (Mdn= 100%, n=16) than for control group students (Mdn= 60%, n=16) on the post-assessment exam (U= 73.5, p < 0.042). The interventional group had a higher mean of ranks at 19.91 compared to the control group at 13.09, a 66% difference, indicating an increased score in the interventional group compared to the control group. The  effect size utilizing rank-biserial correlation showed a moderate effect size (rrb = 0.426), suggesting that the magnitude of difference between the control and interventional groups is moderate. (Table 1)

**Table 1:** Mean competency scores, Mann-Whitney U tests, and effect size from the control and interventional groups

Scores	Control Group (n=16)		Interventional Group (n=16)			
	Mean ± SD	Mean of Ranks	Mean ± SD	Mean of Ranks	p-value	Effect Size (*r_rb_*)
Diagnostic Accuracy	58.75% ± 1.77	13.09	85% ± 0.93	19.91	< 0.042*	0.426^†^
Knowledge Acquisition	46.25% ± 1.19	11.56	76.25% ± 1.11	21.44	< 0.031*	0.617^††^

* p < 0.05, Mann-Whitney U test

^†^ Rank-biserial correlation 0.4-0.6, moderate effect size

^††^ Rank-biserial correlation > 0.6, strong effect size

### Knowledge Acquisition

All 32 participants also completed the post assessment exam for knowledge acquisition. The average score in the control group was 46.25% (SD = 1.19) and the average score was 76.25% (SD= 1.11) in the interventional group. (Table 1) The Shapiro-Wilks test was performed and showed that the distribution of scores deviated significantly from normality in the control (W= 0.89, p-value < 0.05) and interventional groups (W= 0.84, p-value < 0.01). Due to the substantial skewness of the data, a non-parametric Mann-Whitney U test was used for analysis. The analysis also indicated that knowledge acquisition was significantly improved for multisensory students (Mdn= 80%, n=16) than for control group students (Mdn= 50%, n=16) on the post-assessment exam (U= 49, p < 0.031). The interventional group had a higher mean of ranks at 21.44 compared to the control group at 11.56, a 54% difference, indicating an increased score in the interventional group compared to the control group. The effect size utilizing rank-biserial correlation showed a strong effect size (rrb = 0.617), suggesting that the magnitude of difference between the control and interventional groups is substantial. (Table 1)

## Discussion

The primary objective of this study is to determine the effectiveness of multisensory educational techniques for improving overall cardiac auscultation competency in medical students. Competency was measured using two categories: diagnostic accuracy and knowledge acquisition. The first category assesses participants' diagnostic accuracy. The results support that the multisensory approach significantly improves the identification of heart sounds by participants. Improvements in diagnostic accuracy alone could have positive implications for patient outcomes. Cardiac auscultation improvements may allow for earlier intervention of cardiac abnormalities before progressing to a cardiac pathology. This idea is reflected in the study by Compostella and colleagues, where the study found that nurses' ability to accurately detect abnormal heart sounds improves patient outcomes in post-cardiac rehabilitation.^19^

The same logic may extend into other healthcare settings, such as primary care fields. In primary care, physicians see patients routinely, allowing for early detection of pathologies. However, with 80% of new physicians unable to identify abnormal heart sounds, early cardiac pathology detection may be reduced in this setting.^5^ Additionally, the broadness of the primary care patient population may lead to physicians lacking cardiac auscultation expertise, thus it is important to provide more effective training starting in medical school. A multisensory approach to education may elevate diagnostic accuracy and lead to earlier detection of abnormal heart sounds.

The secondary category assessed, knowledge acquisition, determined if participants could explain the pathophysiology of the heart sound identified. The results significantly suggest that multisensory learning benefits knowledge acquisition in the interventional group when compared to the control group. Although understanding pathophysiology may not directly benefit auscultation of the heart, this may help reduce auscultation anxiety of healthcare professionals. A study by Dogru and Aydin concluded that nursing students (n=36) who received high fidelity simulator teaching reported improved knowledge, improved cardiac auscultation skills, and decreased anxiety when compared to nursing students (n=36) who received traditional teaching.^20^ Additionally, understanding the pathophysiology may allow clinicians to more effectively treat pathologies without unnecessary cardiology consultations. Future research may provide more insight into the correlation between understanding disease mechanisms and the quality of treatment.

The moderate effect size observed in diagnostic accuracy within this study suggests that the novel multisensory training program has a tangible, positive impact on students' ability to accurately identify heart sounds, though the effect is not overwhelmingly large. This outcome implies that while the program significantly enhances diagnostic skills, its effectiveness could be potentially maximized by addressing other influencing factors or by integrating it with traditional learning methods for a more comprehensive approach. This is supported by D’Anonti and colleagues suggesting that learning strategies such as mind maps using colors and pictures can potentially enhance the longer-term memory of medical students.^22^ On the other hand, the strong effect size in knowledge acquisition underscores the substantial benefits of the multisensory approach in deepening students' understanding of pathophysiology. This significant effect size strongly supports the adoption of the multisensory training program for medical education, as it evidently fosters a deeper comprehension of the material, potentially leading to more adept and informed clinical decision-making. Together, these findings advocate for the integration of multisensory learning strategies in medical curricula to enhance both practical diagnostic skills and theoretical knowledge, although the approach may need to be tailored to maximize its effectiveness across different learning objectives.

After analyzing diagnostic accuracy and knowledge acquisition, our findings demonstrate that multisensory learning can be more effective than traditionally used uni-sensory training paradigms. To our knowledge, this is the first study to integrate auditory, visual, and tactile stimuli together when teaching cardiac auscultation. One advantage of such multisensory learning is that it can engage individuals with different learning styles such as visual learners, auditory learners, kinesthetic learners. This allows for information from different modalities to be easily organized into short-term memory and used to build long-term memory.

Moreover, the simplicity of our multisensory learning intervention offers a practical advantage that can facilitate its implementation in medical education programs. Unlike other interventions, such as Harvey, our intervention is cost effective by mainly requiring only a 3D model of a simulated heart with synchronized heart sounds, and a surface to tap on, which can be easily obtained and replicated across various medical institutions. Lastly, our protocol is straightforward and easy to follow, enabling students and instructors to participate in the learning process without prior experience or extensive training.

### Limitations

Our study acknowledges several limitations. One limitation is the differences in participant engagement between groups. Traditional education may have been perceived as more familiar, contributing to a decrease in participant engagement. Conversely, the novelty of 3D simulation and the instructions to tap along may have increased participant engagement. Previous studies have considered these ideas and suggested that the novelty of simulation may affect overall engagement in educational lessons.^21^ These considerations may partially explain the wide differences in overall competency between groups.

Several confounding variables might have influenced the study outcomes, adding complexity to the interpretation of the results. Among these variables, participant characteristics such as learning styles and healthcare experience are particularly important. Although in theory a multisensory approach would eliminate this, some participants might have preferred visual or auditory learning, while others may have been more inclined towards tactile learning. These individual preferences could have affected their performance and engagement with the educational module, irrespective of the intervention. Additionally, even though cardiac auscultation experience was screened out, as all participants were first-year medical students, they all have had some kind of previous healthcare exposure. Therefore, participants with more prior miscellaneous healthcare experience might have had an advantage in the study, as they could have been more familiar with the techniques and terminology involved. These individual differences in learning styles and experience could have influenced the observed improvements, making it challenging to attribute the results solely to the multisensory teaching approach.

Furthermore, considering that the participants were first-year medical students just starting their medical education journey, they might have experienced heightened anxiety levels during the study, affecting their ability to concentrate and perform well during the study. This confounding variable also ties into the potential for the Hawthorne effect to skew the results. The combination of the participants knowing they are being watched and already elevated anxiety to perform well may have influenced the outcomes.

Another source of bias was the preliminary nature of the study. The limited sample size and small number of multiple-choice questions may have restricted the generalizability of the results. Consequently, these findings should be cautiously interpreted. Lastly, our study was conducted at a single institution, reflecting the outcomes of only one institution's medical students.

In summary, while our study has shown promising results, it is important to acknowledge the various limitations, including differences in participant engagement, confounding variables such as individual learning styles and healthcare experiences, potential anxiety, the Hawthorne effect, and the preliminary nature of the study. Although our results should be interpreted with caution, they offer a solid foundation for future research in multisensory education across various skill sets, including pulmonary auscultation. By involving a larger pool of medical students from different institutions, further insights into the validity and generalizability of this clinical education approach can be gained.

## Conclusion

In conclusion, our study found that using multisensory education in cardiac auscultation training significantly improved participants' ability to identify heart sounds and explain their findings. These results suggest that adopting a multisensory approach in medical education can enhance healthcare provider competency and lead to improved patient outcomes. Future research should examine the generalizability of these findings with larger more diverse sample sizes across multiple medical institutions and explore the effectiveness of multisensory education in teaching other clinical skills. Since our study was preliminary in nature, it is crucial to interpret our findings cautiously due to the lack of control over extraneous and confounding variables. Nevertheless, our study brings attention to the potential benefits of multisensory education in bridging the gap between traditional teaching methods and expensive simulation tools, ultimately improving patient healthcare.

### Acknowledgments

The research team would like to acknowledge Mr. Allen Ecker for his support of this study and for providing access to the tools to make this research possible and Dr. Robert Steer for his advice regarding statistical analysis.

### Conflicts of Interest

The authors declare they have no conflicts of interest.
